# A Metal Chelator as a Plasmonic Signal‐Generation Superregulator for Ultrasensitive Colorimetric Bioassays of Disease Biomarkers

**DOI:** 10.1002/advs.201800295

**Published:** 2018-05-21

**Authors:** Qian Zhao, Jiafang Piao, Weipan Peng, Jun wang, Weichen Gao, Xiaoli Wu, Hanjie Wang, Xiaoqun Gong, Jin Chang, Bingbo Zhang

**Affiliations:** ^1^ School of Life Sciences Tianjin Engineering Center of Micro‐Nano Biomaterials and Detection‐Treatment Technology Tianjin University Tianjin 300072 China; ^2^ Institute of Photomedicine Shanghai Skin Disease Hospital The Institute for Biomedical Engineering and Nano Science Tongji University School of Medicine Shanghai 200443 China

**Keywords:** chelating capability, enzyme‐based assays, ethylenediamine tetraacetic acid disodium salt, operation stability, signal generation

## Abstract

Enzyme‐based assays have been widely applied in clinical diagnosis for decades. However, the intrinsic limitations of enzymes, such as low operation stability, mediocre sensitivity, and high cost in production and purification, heavily constrain their detection application. Here, an enzyme‐free assay is reported that relies on the strong chelating capability of ethylenediamine tetraacetic acid disodium salt (EDTA•2Na, the chelator) for Au^3+^ ions, in which the cheap EDTA•2Na labeled by targeting moieties can selectively regulate the growth of plasmonic gold nanoparticles (AuNPs) at the target site subjecting to the concentration of analyte in samples. Independent of ambient temperature and unstable H_2_O_2_, EDTA•2Na perform superregulation in AuNPs plasmonic signal generation with distinct tonality and outstanding reliability. Upon integrating with silica nanoparticles as the signal amplifying platform, EDTA•2Na‐regulated bioassay can lead to detection‐sensitivity enhancements exceeding three orders of magnitude in protein detection, compared with the gold‐standard assay. The limit of detection of the HBsAg and alpha fetoprotein (AFP) pushes down to 2.6 × 10^−15^ and 2.5 × 10^−19^ g mL^−1^, respectively. EDTA•2Na‐regulated bioassay is also challenged in the clinical serum sample detection and a good consistency is found with the chemiluminescence immunoassay method in clinics.

## 1 Introduction

Developing ultrasensitive, quantitative, and cost‐effective methodologies for the detection of disease‐related low‐abundance biomarkers is becoming an unstoppable trend and should be seen as an exciting challenge.^1^ Enzyme‐based colorimetric assays are widely recognized as such kind of techniques around the world for clinical detections, thanks to their easy operation, straightforward readout, and good practicality.^2^ However, their detection performances are directly dependent on the enzymes' catalytic activity, which inhibits many critical applications such as early screening and detection of cancer biomarkers.^3^

Enzymes (e.g., horseradish peroxidase and alkaline phosphatase) as the signal generators by converting their substrates into colored products are broadly used in enzyme‐based colorimetric assays, such as enzyme‐linked immunosorbent assay (ELISA), immunohistochemistry, and western blot.^4^ However, their shortcomings, including narrow thermophilic scope, poor stability, and high cost (Table S1, Supporting Information), seriously pull down their detection performance and restrict their application in unfriendly circumstances.^3^ Particularly, the catalytic activity of enzymes appears to have been far from enough to satisfy the incremental demands of early disease screening compared to other technologies such as those based on fluorescence and plasmonics.^5^ Ongoing attempts have been carried out to improve the sensitivity of enzyme‐based colorimetric assays, for instance, immobilizing multiple enzymes on nanocarries to amplify the color signals,^6^ or the emerging plasmonic ELISA to further enlarge the signal intensity through in situ production of colored AuNPs as enhanced signal reporters.[[qv: 4h,6a,7]] Instead of enzyme substrates, the biocatalysis of the enzyme label is associated to the growth of AuNPs to obtain blue‐ or red‐colored solutions in the presence or absence of the analyte, respectively.[[qv: 4h,7c]] Meanwhile, nanoparticles possess intrinsic peroxidase‐like activity have received considerable interest, which were similar with the natural peroxidases, for example, Fe_3_O_4_ nanoparticles,^8^ Pd−Ir nanoparticles,^9^ cubic Au@Pt NPs,^10^ and gold nanoclusters.[[qv: 5b]] The fact opens novel applications of these nanoparticles for immunoassay detections. However, these improved techniques either still largely depend on the performance of enzymes without breaking their intrinsic limitations, or need complicate preparation with ligands to help improve the stability but easy abscission.^3^ There is still an urgent need of novel schemes and protocols for substituting enzymes for the biomarkers' detection.

Herein, we report an enzyme‐free signal generation approach to thoroughly get rid of enzymes with significantly improved detection performance including enhanced detection sensitivity and excellent tolerance with the situated testing environments. In this technique, the signals come from the generated colored AuNPs without the catalysis of enzymes but with the regulation of a metal chelator. By leveraging on the metal sequestration of a complexing agent to dominate gold nanoparticle generation (dubbed Scadge), ethylenediamine tetraacetic acid disodium salt (EDTA•2Na), the chelator, is found to be a fine‐tuner in controlling the color or the tonality of AuNPs. Particularly, the tonality of the generated AuNPs is highly correlated with the concentration of analyte in the sample when EDTA•2Na is labeled by antibodies. By utilizing its extraordinary chelating force with metal ions, EDTA•2Na can lock Au^3+^ fiercely and prevent its reduction to AuNPs. Inspired by this Scadge tactics, EDTA•2Na‐regulated signal generation strategy is performed on 96‐well plates to mimic ELISA for the analysis of HBsAg and alpha fetoprotein (AFP). The proposed diagnostic mode is termed as Scadge‐Diag in this study. Furthermore, silica nanoparticles (SiO_2_ NPs) are employed as signal amplification nanoplatforms through carrying a parade of EDTA•2Na on their surfaces to achieve ultrasensitive detection.

**Figure 1:**
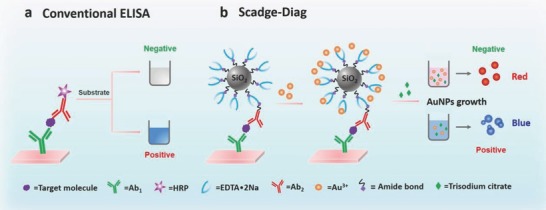
Schematic representation of conventional sandwich ELISA format and Scadge‐Diag bioassay performed in 96‐well polystyrene (PS) plates. a) In the conventional colorimetric ELISA, enzymatic biocatalysis dominates the color change of substrate. b) In the Scadge‐Diag bioassay, leveraging on the metal sequestration of a chelating agent (EDTA•2Na) for dominating gold nanoparticle generation (Scadge), EDTA•2Na substituting enzyme is employed for bioassay and this detection is enhanced through a silica nanocarrier as a signal amplifier.

The schematics of conventional ELISA and our proposed Scadge‐Diag are shown in **Figure** **1**. In the conventional ELISA, enzymes enable their conversion of substrates to be colored products, and the intensity of the colored solution is quantified by measuring the absorbance with a plate reader. In our Scadge‐Diag approach, enzyme is replaced by a very stable small molecule, and crucially, antibody‐labeled EDTA•2Na can act as specific superregulators to selectively control the generation of AuNPs, achieving a particular tonality for quantitative plasmonic assay.

## 2 Result and Discussion

### 2.1 Tonality Tuning of AuNPs at Physiological Temperature

**Figure 2:**
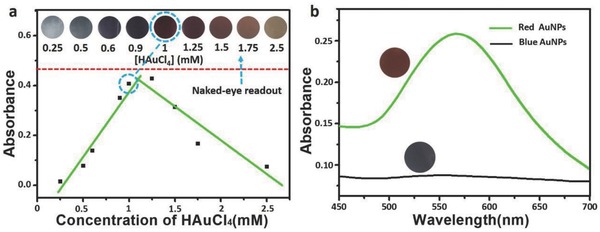
Synthesis of AuNPs at physiological temperature and their tonality characterizations. a) Demonstration of generated AuNPs with different colors dependent on the concentrations of 100 µL of HAuCl_4_ from 0.25 × 10^−3^ to 2.5 × 10^−3^ m in 100 µL of 2 × 10^−3^ m of trisodium citrate solution at 37 °C. The absorbance values of the solution at 550 nm are plotted against varying concentrations of HAuCl_4_. b) UV–vis spectra of AuNPs generated with HAuCl_4_ concentrations of 1 × 10^−3^ m (green line) and 0.5 × 10^−3^ m (black line), respectively. Inset: Photographs of the corresponding AuNPs solutions.

In this study, efficient synthesis of AuNPs at 37 °C was achieved by reduction of Au^3+^ in the presence of trisodium citrate. Particularly, the color or the tonality of the resultant AuNPs was found to be controlled by the concentration of Au^3+^. As displayed in **Figure** **2**a, the color of AuNPs gradually changes from blue to red with the increase of Au^3+^ and the corresponding absorbance values at 550 nm (*A*_550nm_) plotted against concentrations of Au^3+^ are also provided with a good linear scope at the range from 0.25 × 10^−3^ to 1 × 10^−3^ m, which favors quantitative calculation for biodetection.

In order to disclose the cause of gradient tonality of AuNPs, transmission electron microscopy (TEM) technique was used to observe the size and morphology of the generated AuNPs. Interestingly, the resultant nanoparticles tend to be aggregated with ill‐defined morphologies at the lower concentrations of Au^3+^, giving rise to a blue solution (Figure S1a–d, Supporting Information). While at higher Au^3+^ concentration (e.g.,1.0 × 10^−3^ m), uniform dispersed AuNPs with nonaggregated and spherical morphologies were obtained and turned the solution red (Figure S1e, Supporting Information). Herein, two representative states of AuNPs generated with 0.5 × 10^−3^ and 1.0 × 10^−3^ m of Au^3+^ are demonstrated via two distinct colors and UV–vis spectra (Figure 2b).

The fact that the color or the tonality of the generated AuNPs is directly associated with the amount of the added Au^3+^ ions, inspires us leveraging on the sequestrating or consuming Au^3+^ ions by an antibody‐labeled chelator, we can selectively modulate the concentration of Au^3+^ ions subjecting to the analyte in the sample solution and further in situ regulate AuNPs generation, as such, a definite color of as‐generated AuNPs can be obtained in a specific case for bioassays.

### 2.2 Validating the Efficacy of EDTA•2Na on Sequestration of Au^3+^ Ions

**Figure 3:**
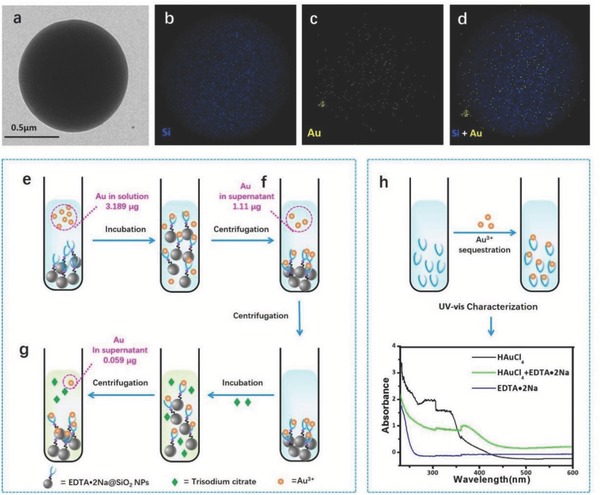
Elemental distribution analysis of EDTA•2Na@SiO_2_‐Au chelates and investigation of the efficacy of EDTA•2Na on sequestering Au^3+^ ions by ICP‐MS technique. a) TEM image of EDTA•2Na@SiO_2_‐Au chelates. b–d) EDX mapping image of EDTA•2Na@SiO_2_‐Au chelates (blue = Si, yellow = Au). 500 µL of HAuCl_4_ (2 × 10^−3^ m) were added to EDTA•2Na@SiO_2_ solutions for chelation and detected through X‐ray (EDX) mapping. e,f) 500 µL of HAuCl_4_ (0.00638 g L^−1^) were added to EDTA•2Na@SiO_2_ solutions for chelation and after centrifugation the changes in Au contents were detected through ICP‐MS. g) The precipitates of EDTA•2Na@SiO_2_‐Au chelates obtained from (b) were redispersed in water and further incubated with 500 µL of trisodium citrate solution (2 × 10^−3^ m). The contents of Au in supernatant after centrifugation were detected through ICP‐MS. h) UV–vis spectra of HAuCl_4_, EDTA•2Na, and their mixture.

The coordination interaction between EDTA•2Na and Au^3+^ ions was investigated by energy dispersive X‐ray (EDX) mapping, as shown in **Figure** **3**a–d, the elemental distribution of Si (blue) in the EDTA•2Na@SiO_2_‐Au chelates could be observed in the entire nanoparticle, while Au (yellow) was distributed on the surface of the SiO_2_ nanoparticle, suggesting the coordination interaction between EDTA•2Na and Au^3+^ ions on the EDTA•2Na@SiO_2_. Then, the exact binding quantity of Au^3+^ ions was further characterized by inductively coupled plasma mass spectrometry (ICP‐MS) technique as illustrated in Figure 3e–g. It showed that Au^3+^ ions were effectively captured by the EDTA•2Na molecules which were aforehand anchored on the surface of SiO_2_ NPs. The content of Au^3+^ ions was found to be reduced from 3.189 to 1.11 µg upon chelation (Figure 3e,f) with a chelating efficiency of one 500 nm sized EDTA•2Na@SiO_2_ nanoparticle capturing about 1108 Au^3+^ ions (Equations (S1)–(S3), Supporting Information). More importantly, the locked Au^3+^ ions by EDTA•2Na@SiO_2_ were hardly pulled out by freshly added trisodium citrate in the purified EDTA•2Na@SiO_2_‐Au^3+^ chelates with only 1.85% of the original Au^3+^ ions decoupled (Figure 3g), suggesting the strong chelating ability of EDTA•2Na for Au^3+^ ions as intended. Notably, UV–vis spectroscopy measurements further confirm the chelating reaction (Figure 3h). The characteristic absorption peak of ([AuCl_4_]^−^) at 330 nm in HAuCl_4_ redshifts to 360 nm in the [AuCl_4_]^−^ + EDTA•2Na mixed sample. This shift could be induced by the electronic transition from Au^3+^ to EDTA•2Na.^11^ The above results clearly show that EDTA•2Na can effectively captured Au^3+^ with negligible effect by trisodium citrate.

### 2.3 Validating the Efficacy of EDTA•2Na on Scadge

**Figure 4:**
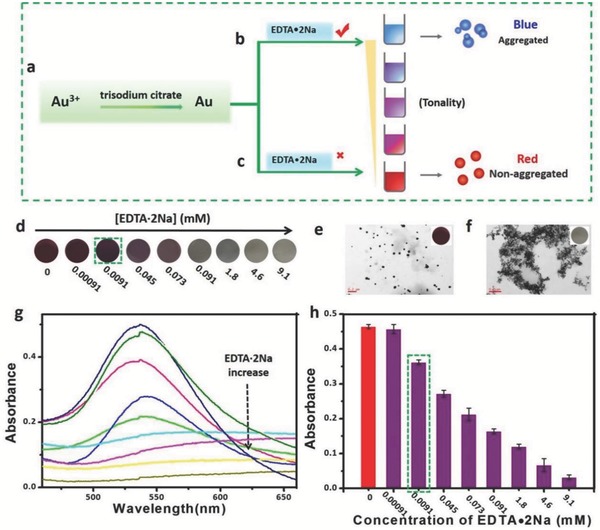
Investigation of the efficacy of EDTA•2Na on Scadge. a–c) An illustration of EDTA•2Na‐regulated AuNPs generation in the solution of trisodium citrate at 37 °C in the b) presence and c) absence of EDTA•2Na. d) Photograph showing AuNPs with different colors from red to blue regulated by varying concentrations of EDTA•2Na from 0.00091 × 10^−3^ to 9.1 × 10^−3^ m, plus no EDTA•2Na involved. TEM images of as‐prepared AuNPs in e) dispersed with 0.00091 × 10^−3^ m EDTA•2Na and f) aggregated formats with 9.1 × 10^−3^ m EDTA•2Na. g) UV–vis spectra of AuNPs prepared with different EDTA•2Na concentrations from 0.00091× 10^−3^ to 9.1 × 10^−3^ m, and h) their corresponding quantitative absorbance values at 550 nm. All experiments, unless otherwise noted, were performed in a mixed solution containing 1 × 10^−3^ m of Au^3+^ ions and 2 × 10^−3^ m of trisodium citrate at 37 °C.

Considering the strong Au^3+^ sequestering capability of EDTA•2Na, we hypothesized that EDTA•2Na can be used to control the generation and therefore the resultant tonality of AuNPs. In this proposal, we introduced EDTA•2Na during the AuNPs formation at 37 °C and found the amount of EDTA•2Na was highly linked to the growth of AuNPs to form blue‐ or red‐colored solutions (**Figure** **4**a–c). Figure 4d showed the capability of EDTA•2Na in color tuning of AuNPs from red to blue in a concentration range between 0.00091 × 10^−3^ and 9.1 × 10^−3^ m of EDTA•2Na. In detail, in a certain amount of Au^3+^ ions solution, with lower concentration of EDTA•2Na, namely less Au^3+^ ions chelated, thus more free Au^3+^ ions were reduced to AuNPs, resulting in well dispersed, quasi‐spherical, nonaggregated wine‐red AuNPs in solution (Figure 4a,c,e). Nevertheless, in the higher concentration of EDTA•2Na solution, more Au^3+^ ions are consumed, resulting in reduced feed and slow kinetics of crystal growth, so tiny and aggregated AuNPs are generated with blue color or colorless as displayed in Figure 4a,b,f. The UV–vis spectra of AuNPs (Figure 4g,h) further reveal that the *A*_550nm_ value is negatively correlated with the concentration of EDTA•2Na. It is worth noting that a tiny amount of EDTA•2Na (0.0091 × 10^−3^ m) still has good capability in dominating generation of AuNPs, suggesting ultrasensitivity of EDTA•2Na as a superregulator in Scadge.

### 2.4 Development and Validation of EDTA•2Na‐Regulated Scadge‐Diag Immunoassay

**Figure 5:**
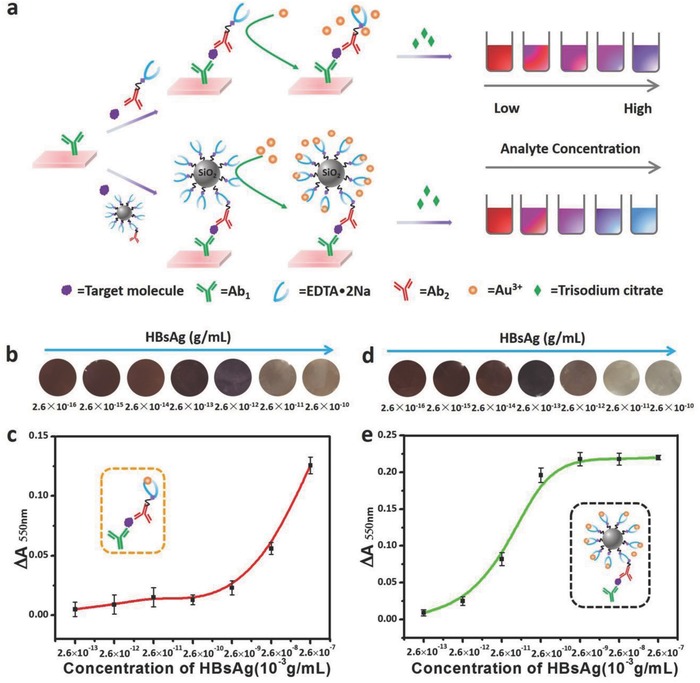
Development and validation of EDTA•2Na‐regulated Scadge‐Diag immunoassay in HBsAg‐exampled ultrasensitive detection. a) Schematic illustration of Scadge‐Diag and SiO_2_ NPs‐amplified Scadge‐Diag. b,d) Practical detection of HBsAg by naked‐eye visualization and c,e) plotting absorbance values of AuNPs collected at 550 nm versus varying concentrations of HBsAg based on b,c) Scadge‐Diag and d,e) SiO_2_ NPs‐amplified Scadge‐Diag, respectively. Error bars indicate the standard deviation of three independent measurements.

After demonstrating the EDTA•2Na‐based regulation of AuNPs generation, namely the Scadge, we further designed the Scadge‐Diag immunoassay platform with EDTA•2Na as the signal fine‐tuner for HBsAg detection on 96‐well plates. Before bioassays, we used UV−vis absorption spectrophotometer for the characterization of the antibody bioconjugation. As shown in Figure S2 in the Supporting Information, the characteristic absorption peak at 280 nm of Ab_2_ was observed in the Ab_2_‐EDTA•2Na@SiO_2_ bioconjugates, indicating the successful conjugation of Ab_2_ onto the surface of EDTA•2Na@SiO_2_ NPs. As shown in **Figure** **5**a, the HBsAg is first captured by the Ab_1_ immobilized on the polystyrene (PS) plate and further recognized by EDTA•2Na‐Ab_2_ bioconjugate, forming a sandwich immunocomplex. Like conventional sandwich ELISA, more targets in the sample cause higher amount of EDTA•2Na‐Ab_2_ bioconjugates depositing at the bottom. As such, more Au^3+^ ions are seized by EDTA•2Na‐Ab_2_ bioconjugates, leading to aggregated blue or colorless AuNPs solution. Conversely, if target molecule is absent or less, none or less EDTA•2Na exists for coordinating Au^3+^ ions, resulting in wine‐red AuNPs dispersions.

The tonality of the generated AuNPs can be qualitatively assessed by naked eyes or quantitatively determined by a plate reader. In order to quantitatively acquire the contents of analytes in the samples, herein, we measured the change of absorbance values (Δ*A*_550nm_, Δ*A*_550nm_ = *A*_0(550nm)_ − *A*_1(550nm)_, *A*_0(550nm)_ represents the absorbance value at 550 nm of the blank experiment, *A*_1(550nm)_ represents the absorbance value at 550 nm of the experimental sample). Obviously, the Δ*A*_550nm_ value was positively correlated with the concentration of the target in the sample (Figure 5b,c).

Moreover, SiO_2_ NPs were introduced as carriers to largely load the EDTA•2Na on the surface (Figure 5), so as to significantly improve the detection sensitivity. The influence of the SiO_2_ NPs size on detection sensitivity was investigated and results showed that 500 nm sized SiO_2_ NPs allowed highest sensitivity among the other three sizes of 100, 300, and 1000 nm (Figure S3, Supporting Information). The dynamic light scattering measurement showed that the SiO_2_ NPs (coded as 500 nm for an easy recognition) homogeneously dispersed in the solution and the real size was 561.3 ± 48 nm. Larger SiO_2_ NPs with more EDTA•2Na loading on the surface enables higher sensitivity. But this trend stops at 1000 nm, mainly due to the steric‐hindrance effect which impedes the immune reactions.

Figure 5b–e shows the detection performance of Scadge‐Diag and SiO_2_ NPs‐amplified Scadge‐Diag strategy. It displayed good gradient tonality of the generated AuNPs solution dependent on the concentration of the targeting antigens in the samples in both cases (Figure 5b,d). Parallel tests further disclosed that SiO_2_ NPs‐amplified Scadge‐Diag strategy had higher detection sensitivity as intended with the limit of detection (LOD) of HBsAg about 2.6 × 10^−15^ g mL^−1^ (Figure 5d,e) compared with that about 2.6 × 10^−13^ g mL^−1^ by Scadge‐Diag (Figure 5b,c). Notably, the LOD was found about over 10^3^‐fold lower than that determined by conventional ELISA (Figure S4, Supporting Information).

### 2.5 Reliability Assessment of Scadge‐Diag Immunoassay

**Figure 6:**
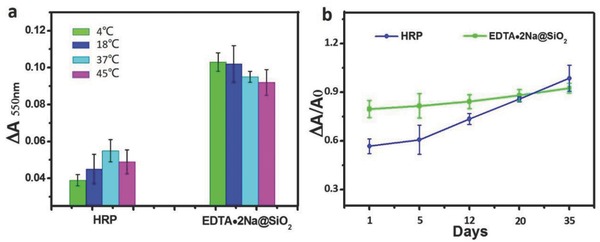
Stability study of HRP and EDTA•2Na@SiO_2_ in immunoassay. a) Δ*A* values in detection of HBsAg as an example (2.6 × 10^−12^ g mL^−1^) at different operating temperatures (4, 18, 37, and 45 °C, respectively) based on HRP and EDTA•2Na@SiO_2_. b) Plotting Δ*A*_550nm_/*A*_0(550nm)_ values of AuNPs versus varying storage periods (1, 5, 12, 20, and 35 d) at 4 °C based on HRP and EDTA•2Na@SiO_2_.

The thermal and long‐term stability of Ab_2_‐EDTA•2Na@SiO_2_ are highly important for their clinical applications, especially in the stringent conditions and resource‐constrained areas. As shown in **Figure** **6**a, the ∆*A*_550nm_ values of the generated AuNPs almost kept consistent without obvious fluctuations in Scadge‐Diag system at different operating temperatures (4, 18, 37, and 45 °C, respectively), indicating good resistance of Ab_2_‐EDTA•2Na@SiO_2_ to heat. However, the Δ*A*_550nm_ values in the Ab_2_‐horse radish peroxidase (HRP) experimental group changed significantly as the temperature increases because the catalytic activity of HRP is highly dependent on the working temperature.

The storage stability of Ab_2_‐EDTA•2Na@SiO_2_ was further investigated after varying storage periods (1, 5, 12, 20, and 35 d) at 4 °C. The Δ*A*_550nm_/*A*_0(550nm)_ value based on the Ab_2_‐HRP conjugates increased obviously in a slope manner with the increasing storage time (Figure 6b), due to the easy denaturation and digestion of enzymes.^9^ Fortunately, the Δ*A*_550nm_/*A*_0(550nm)_ d curve obtained by Ab_2_‐EDTA•2Na@SiO_2_ appears flat, indicating good stability of EDTA•2Na, even after a long‐term storage. Owing to the unique features of EDTA•2Na, such as the small molecular weight, stable structure and much low cost, EDTA•2Na provides a wider thermophilic scope and long‐term storage stability, and outstanding reliability in Scadge‐Diag system.

### 2.6 Calibration Curve establishment and Specificity Evaluation of Scadge‐Diag Immunoassay

**Figure 7:**
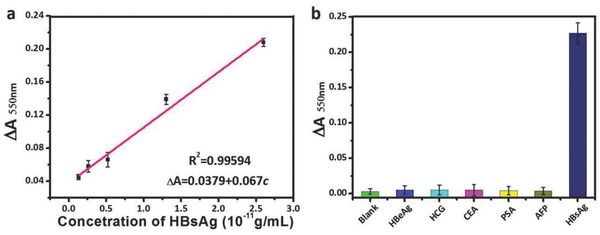
Calibration curve establishment and detection specificity evaluation. a) Calibration curve of HBsAg using SiO_2_ NPs‐amplified Scadge‐Diag. b) Specificity tests: Δ*A* at 550 nm measured from various interfering biomarkers including 25 ng mL^−1^ HBeAg, 52 U mL^−1^ HCG, 72 ng mL^−1^ CEA, 50 ng mL^−1^ PSA, 10 ng mL^−1^ AFP, and 5 ng mL^−1^ HBsAg. Error bars show the standard deviations of three independent measurements.

Before clinical human sample detection of HBsAg, a calibration curve was obtained by plotting ∆*A*_550nm_ of the generated AuNPs solutions against various concentrations of HBsAg (**Figure** **7**a). It showed a good linearity between the ∆*A*_550nm_ and the concentration of HBsAg from 1.3 × 10^−12^ to 2.6 × 10^−11^ g mL^−1^. The resultant calibration equation is Δ*A*_550nm_ = 0.0379 + 0.06*c*, with a linear regression coefficient of 0.99594. We also evaluated the detection specificity of SiO_2_ NPs‐amplified Scadge‐Diag system by introducing HBeAg, human chorionic gonadotropin (HCG), carcinoembryonic antigen (CEA), prostate specific antigen (PSA), and AFP as the control groups. As shown in Figure 7b, the negligible changes at ∆*A*_550nm_ in these control groups suggested good specificity of Scadge‐Diag system in biospecific recognition. In addition, besides HBsAg, AFP as a key liver cancer biomarker was also detected by using SiO_2_ NPs‐amplified Scadge‐Diag technique. Results showed that it also enabled a dramatic low LOD of 2.5 × 10^−19^ g mL^−1^ as well as a good linear relationship (Figure S5, Supporting Information).

### 2.7 HBsAg Detection in Clinical Serum Samples

**Figure 8:**
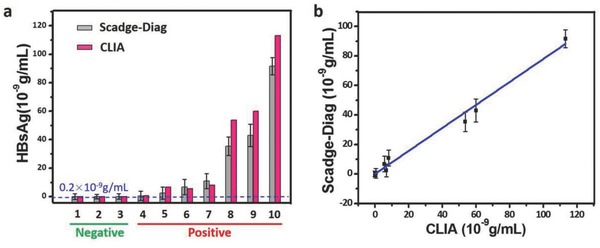
Detection of HBsAg in human serum samples by SiO_2_ NPs‐amplified Scadge‐Diag and CLIA. a) Detection results of HBsAg in negative and positive samples by Scadge‐Diag system and CLIA method, respectively. Error bars indicate the standard deviation of three independent measurements. b) Correlation analysis between Scadge‐Diag and CLIA in quantification of HBsAg (*r* = 0.9884).

After establishing Scadge‐Diag system, SiO_2_ NPs‐amplified Scadge‐Diag immunoassay was applied to determine the contents of HBsAg in clinical serums of patients suffering from HBsAg infection. In this study, three HBsAg‐negative samples and seven HBsAg‐positive samples were collected and diluted by phosphate buffered saline (PBS) buffer respectively before double‐blind detection. On the basis of the criteria set by clinical detection protocols, it could be considered positive when the concentration of HBsAg is higher than 0.2 × 10^−9^ g mL^−1^. We detected the human samples by Scadge‐Diag based on the above established calibration curve (Figure 7a), and the acquired results were further compared with those determined by chemiluminescence immunoassay (CLIA) method in clinics (**Figure** **8**a). It is worth celebrating that these two methods have good correlation with a correlation coefficient *r* = 0.9884 (Figure 8b). Although the number of clinical serum samples is limited in this present study, the preliminary results demonstrate Scadge‐Diag is compelling, and proposing a new signal generation mechanism through a metal chelator with a workable reality.

Conventional enzymes‐based assay, such as ELISA, though it has made strides in the advancement of bioassays, suffers from the shortcomings of vulnerable natural enzymes, including low operational stability, high costs in preparation and purification, and difficulties in recovery and recycling. Moreover, the activities of natural enzymes are easily to be affected by environmental conditions such as pH, temperature, and chemical compounds, seriously blocking its utilizations in colorimetric sensing technics.[[qv: 3a,c,12]] In this study, a novel enzyme‐free sandwich bioassay with a new signal generation regulation mechanism is demonstrated for ultrasensitive detections of HBsAg and AFP. Capitalizing on the strong sequestration of a complexing agent for dominating gold nanoparticles generation, namely, Scadge, EDTA•2Na is imparted to the current bioassay format. The constructed Scadge‐Diag strategy provides many attractive features for biodetection, such as high sensitivity, good stability, low cost, and particularly good compatibility with the existing infrastructures.

Scadge‐Diag accommodates current ELISA platforms but replacing enzymes by EDTA•2Na. Our results prove that EDTA•2Na is a superregulator on tuning the tonality of the generated AuNPs in the solution of trisodium citrate at 37 °C, due to the extraordinary chelating force of EDTA•2Na with metal ions. Results show that EDTA•2Na can lock Au^3+^ ions fiercely and prevent them being pulled out by trisodium citrate for further reduction to AuNPs (Figure 3). The amount or the tonality of the generated AuNPs was found to be highly dependent on the concentration of EDTA•2Na in the solution (Figure 4).

It makes sense that antibody‐labeled EDTA•2Na can selectively control the tonality of the generated AuNPs, therefore achieving a particular tonality in a specific serum sample. The signal can be sensed by naked eyes and also quantitatively read out by equipments. Also of note, this technology coupled with a nanoamplifier (SiO_2_ NPs) can further improve its detection sensitivity, exceeding those LODs without signal amplification by more than two orders of magnitude and three orders of magnitude than that of conventional ELISA, thanks to the large loading of EDTA•2Na.

The majority of the available evidence in the literature suggests that currently used AuNPs as signal reporters or quenchers for assays are mostly fabricated by reduction of Au^3+^ in the present of trisodium citrate at boiling temperature and with heating reflux equipment for several hours.^13^ This rigorous process obviously does not meet the requirement in an in situ signal‐generation mechanism. Moreover, an effective wavelength, namely an appropriate size and morphology of AuNPs, and their surface should have been well tailored before biomedical detections. Conversely, in this study, the polytropy of AuNPs generated at 37 °C provides a distinct colored mapping, favoring for quantifiable colorimetric detection. This tactic is previously reported in plasmonic ELISA, in which HRP can degrade H_2_O_2_ and further control AuNPs generation in the solution of Au^3+^ ions at 37 °C; however, it still relies on an enzyme.

Herein, Scadge‐Diag assay is found with better detection performance than conventional ELISA in harsh environments, such as high temperatures and long‐term storage (Figure 6). These factors seriously reduce the activity of enzymes in ELISA,[[qv: 4h]] leading to decreased sensitivity or failure in detection due to the inherent vulnerability of enzymes. These shortcomings are effectively circumvented in our Scadge‐Diag platform, thanks to the good stability of EDTA•2Na, regardless of high temperature or long‐term storage. Although only detections of HBsAg and AFP were exemplified for proof‐of‐concept study of Scadge‐Diag in this study, the methodology proposed here could potentially be extended for the detection of any other analyte as long as antibodies or complementary strands of DNA directed against it were available. Hopefully, these features plus much lower cost could facilitate the practical applications of Scadge‐Diag.

## 3 Conclusion

In conclusion, we have demonstrated a high‐performance enzyme‐free assay for protein detection with substantially enhanced sensitivity, low cost, and compatibility with unfriendly testing conditions. By leveraging on the excellent fine‐tuning capability of EDTA•2Na in signal generation, our developed EDTA•2Na‐regulated Scadge‐Diag platform dispels the long‐suffering shortcomings of enzymes thoroughly and shows high consistency with the clinical CLIA method. This assay is validated with solid evidence in the ultrasensitive detection of two kinds of serum proteins in the present work, and it can be easily shifted to detect nearly any other analytes as long as their corresponding targeting moieties found. Importantly, this platform can be directly plugged into the infrastructures in the current biomedical laboratories, therefore making it practically useful and fascinating in clinical diagnostics. We expect that this robust Scadge‐Diag bioassay could find its immediate impacts, especially in those areas that expensive and bulky equipment are not applicable.

## 4 Experimental Section

*Chemicals*: EDTA•2Na and bovine serum albumin (BSA) were purchased from Sigma‐Aldrich. Gold (III) chloride trihydrate (HAuCl_4_·3H_2_O), trisodium citrate, ethylcarbodiimide hydrochloride (EDC•HCl), *N*‐hydroxysuccinimide (NHS), and PBS (pH 7.4) were obtained from Aladdin Industrial Corporation. All of the chemicals, unless mentioned otherwise, were of analytical reagent grade and obtained from the commercial source. Anti HBsAg, anti AFP antibodies (Ab_1_, Ab_2_), AFP antigen, HBsAg antigen, HBeAg antigen, HCG antigen, CEA antigen, and PSA antigen were supplied by Bioscience (Tianjin) Diagnostic Technology Co., Ltd. Commercial hepatitis B surface antigen (HBsAg) enzyme‐linked immunosorbent assay kit was obtained from Shuangying (Shanghai) Biological technology Co., Ltd.

*Synthesis of AuNPs at 37 °C*: AuNPs with varying tonalities were prepared via trisodium citrate reduction at 37 °C. Briefly, 100 µL of different concentrations of HAuCl_4_·3H_2_O (0.5 × 10^−3^, 1 × 10^−3^, 1.2 × 10^−3^, 1.8 × 10^−3^, 2 × 10^−3^, 2.5 × 10^−3^, 3 × 10^−3^, 3.5 × 10^−3^, and 5 × 10^−3^ m) solution was added to the 100 µL of trisodium citrate (4 × 10^−3^ m), respectively. After 1 h incubation, the UV–vis absorption spectra and the absorbance values at 550 nm of the resultant reaction solutions were recorded respectively with an UV–vis spectrophotometer.

*Coordination Chemistry Study of EDTA•2Na with Au^3+^*: The coordination reaction between EDTA•2Na and Au^3+^ was performed on SiO_2_ NPs and verified by ICP‐MS technique. Amine‐functionalized SiO_2_ NPs (SiO_2_‐NH_2_) were synthesized according to the protocol as reported.^14^

*EDTA•2Na‐Anchored SiO_2_ NPs (EDTA•2Na@SiO_2_) Preparation*: Briefly, 0.045 g EDC and 0.043 g NHS were added into 1 mL of EDTA•2Na solution (0.1 mol L^−1^) and incubated for 15 min at room temperature. After that, 500 µL of SiO_2_ NPs (500 nm, 1.5 mg mL^−1^) was added into the above mixture and allowed to react for 12 h under stirring. The purified EDTA•2Na@SiO_2_ was obtained by centrifugation at 10 000 g for 8 min to remove the free EDTA•2Na and by‐products.

*Coordination Chemistry Evaluation of EDTA•2Na with Au^3+^ via ICP‐MS Technique*: Briefly, 500 µL of HAuCl_4_ solution with an initial concentration of 0.00638 g L^−1^ was added to EDTA•2Na@SiO_2_ water solution and incubated for two hours. Then, the mixed solution was centrifuged at 10 000 rpm for 8 min and the supernatant was detected for the content of Au by ICP‐MS. Furthermore, the precipitates were redispersed in 500 µL of fresh trisodium citrate solution (4 × 10^−3^ m). The redispersed solution was then centrifuged again at 10 000 rpm for 8 min and the supernatant was detected for the content of Au by ICP‐MS for checking whether trisodium citrate can pull out the locked Au^3+^ ions or not.

*Efficacy Study of EDTA•2Na on AuNPs Generation*: 20 µL of EDTA•2Na solution with different concentrations (100 × 10^−3^, 50 × 10^−3^, 20 × 10^−3^, 10 × 10^−3^, 1 × 10^−3^, 0.8 × 10^−3^, 0.5 × 10^−3^, 0.01 × 10^−3^, and 0.001 × 10^−3^ m) were added to 20 µL of HAuCl_4_ (2 × 10^−3^ m), respectively, and then 100 µL of freshly prepared trisodium citrate (4 × 10^−3^ m) were added into each of the solution. After incubation for 1 h at 37 °C, the UV–vis absorption spectra and the absorbance values of the generated AuNPs at 550 nm were measured, respectively.

*Preparation of Ab_2_‐EDTA•2Na@SiO_2_ and Ab_2_‐EDTA•2Na Bioconjugates*: EDTA•2Na@SiO_2_ was prepared according to the protocol described above. Antibody‐labeled Ab_2_‐EDTA•2Na@SiO_2_ and Ab_2_‐EDTA•2Na bioconjugates were prepared via EDC/NHS‐mediated coupling reaction between the —COOH groups and the —NH_2_ groups.^15^

*Scadge‐Diag Immunoassay Setup*: The immunological reaction of Scadge‐Diag was conducted in a 96‐well PS plate. Firstly, the 96‐well PS plate was modified by Ab_1_ antibody through incubating with 300 µL of 0.02 mg mL^−1^ Ab_1_ in carbonate buffer at 4 °C overnight. After washing each well of the plate three times with PBS buffer, 300 µL of blocking solution (BSA in PBS, 1 mg mL^−1^) was added and incubated for 1 h at room temperature. Subsequently, each well was washed three times again with PBS buffer, and then 300 µL of HBsAg with varying concentrations (2.6 × 10^−10^ 2.6 × 10^−16^ g mL^−1^) was added to each well. After 1 h incubation, the plate was washed three times. Then, Ab_2_‐EDTA•2Na@SiO_2_ and Ab_2_‐EDTA•2Na bioconjugates were added respectively and incubated at 37 °C for another 1 h. After washing the PS plates three times, trisodium citrate (100 µL, 4 × 10^−3^ m) and freshly prepared HAuCl_4_·3H_2_O (100 µL, 2 × 10^−3^ m) were added to each well of the plate and incubated for 40 min at 37 °C. The generated AuNPs with different colors were photographed and their corresponding absorbance values at 550 nm were measured respectively for plotting. The procedure of detection of AFP was the same with that of HBsAg.

*HBsAg Detection by Clinical ELISA*: HBsAg detection was also conducted by a commercial HBsAg ELISA kit with a detection range from 2.5 × 10^−12^ to 75 × 10^−12^ g mL^−1^. Briefly, 50 µL of HBsAg (0, 2.5 × 10^−12^, 3.75 × 10^−12^, 7.5 × 10^−12^, 15 × 10^−12^, and 30 × 10^−12^ g mL^−1^) was added in the 48‐well plate coated with HBsAg antibody respectively and incubated for 30 min at 37 °C. Subsequently, each well was washed five times with washing liquid, and then 50 µL of HRP‐conjugate reagent was added and incubated for 30 min at 37 °C. After washing the PS plates five times again, 50 µL of chromogen solution A and 50 µL of chromogen solution B were added simultaneously. After incubated for 30 min at 37 °C, 50 µL of stop solution was added and the absorbance values were measured by a microplate reader.

*Stability Study of Scadge‐Diag Platform*: The stability of Scadge‐Diag platform was evaluated at different operating temperatures (4, 18, 37, and 45 °C) and storage time (1, 5, 12, 20, and 35 d) by using detection of HBsAg (2.6 × 10^−12^ g mL^−1^) as an example.

*Clinical Human Sample Analysis*: Human serum samples were obtained from volunteers who signed consent forms before blood collection. All experimental procedures were performed in compliance with the relevant laws and institutional guidelines of Tianjin University. The concentrations of HBsAg antigen in serum samples were determined by our proposed Scadge‐Diag and also by the CLIA method used in clinic for comparison.

## Conflict of Interest

The authors declare no conflict of interest.

## Supporting information

Supplementary
