# Supplementary material for: A Metal Chelator as a Plasmonic Signal‐Generation Superregulator for Ultrasensitive Colorimetric Bioassays of Disease Biomarkers
Source: Adv Sci (Weinh). 2018 May 21;5(7):1800295. doi: 10.1002/advs.201800295 (PMC6051378; doi:10.1002/advs.201800295)
Supplement: Supplementary file 1 — Supplementary [file ADVS-5-1800295-s001.pdf]

Copyright WILEY-VCH Verlag GmbH & Co. KGaA, 69469 Weinheim, Germany, 2018.

ADVANCED SCIENCE
Open Access

Supporting Information

for *Adv. Sci.,* DOI: 10.1002/advs.201800295

A Metal Chelator as a Plasmonic Signal-Generation Superregulator for Ultrasensitive Colorimetric Bioassays of Disease Biomarkers

Qian Zhao, Jiafang Piao, Weipan Peng, Jun wang, Weichen Gao, Xiaoli Wu, Hanjie Wang, Xiaoqun Gong, Jin Chang,* and Bingbo Zhang**

Supporting Information

A Metal Chelator as a Plasmonic Signal-Generation Superregulator for Ultrasensitive Colorimetric Bioassays of Disease Biomarkers†

Qian Zhao,‡[a] Jiafang Piao,‡[a] Weipan Peng,[a] Jun wang,[b] Weichen Gao,[a] Xiaoli Wu,[a] Hanjie Wang,[a] Xiaoqun Gong,*[a] Jin Chang*[a] and Bingbo Zhang*[b]

[a] *School of Life Sciences, Tianjin Engineering Center of Micro-Nano Biomaterials and Detection-Treatment Technology, Tianjin University, Tianjin, 300072, China.*

[b] *Institute of Photomedicine, Shanghai Skin Disease Hospital; The Institute for Biomedical Engineering & Nano Science, Tongji University School of Medicine, Shanghai, 200443, China.*

‡ These authors contributed equally to this work.

†Electronic supplementary information (ESI) available. See DOI:

Correspondence and requests for materials should be addressed to X.G. (email: gongxiaoqun@tju.edu.cn), J.C. (email: jinchang@tju.edu.cn) or to B.B.Z. (email: bingbozhang@tongji.edu.cn)

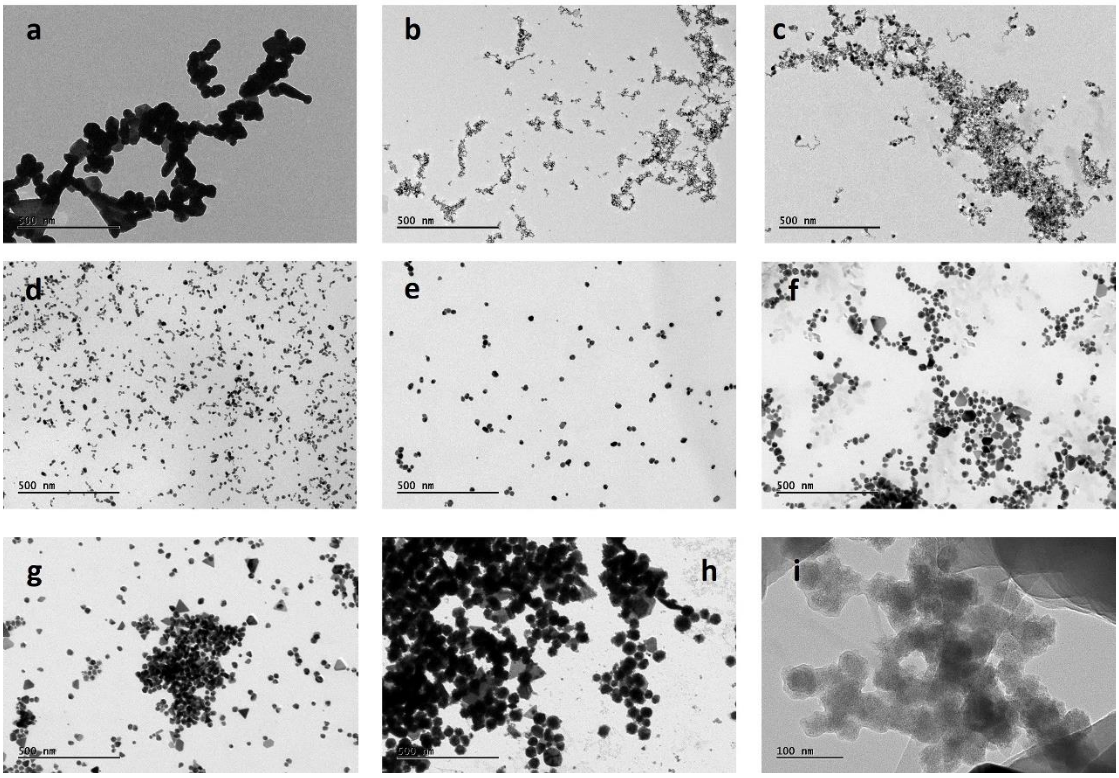

Figure S1. TEM images of AuNPs synthesized with varying concentrations of $HAuCl_4$ in the solution of 2.0 mM trisodium citrate at 37 °C. a-i) The concentrations of $HAuCl_4$ were 0.25 mM, 0.5 mM, 0.6 mM, 0.9 mM, 1 mM, 1.25 mM, 1.5 mM, 1.75 mM and 2.5 mM, respectively.

Calculation of the chelating efficiency of EDTA•2Na@SiO_2 nanoparticles with Au^{3+} ions

Theoretical Models:

$$\mathrm{N}(SiO_2\mathrm{NPs}) = \frac{m(SiO_2\mathrm{NPs})}{\rho(SiO_2\mathrm{NPs}) \times \frac{4}{3}\pi(r_{SiO_2\mathrm{NP}})^3}$$

$$= \frac{0.75mg}{2mg/cm^3 \times \frac{4}{3}\pi(250nm)^3}$$

$$= 5.74 \times 10^{12} \qquad \text{Eqn.1}$$

$$= \frac{m(Au)}{M(Au)} \times N_A = \frac{2.079\mu g}{197g/mol} \times 6.02 \times 10^{23}$$

$$= 6.36 \times 10^{15} \qquad \text{Eqn.2}$$

$$R_{\text{Au on one }SiO_2\text{NP}} = \frac{\text{N(Au)}}{\text{N}(SiO_2\text{NPs})} = \frac{6.36 \times 10^{15}}{5.74 \times 10^{12}} = 1108.01 \qquad \text{Eqn.3}$$

$\text{N}(SiO_2\text{NPs}) = SiO_2\text{NP number}$

$\text{N}(Au) = Au$ number

$R_{\text{Au on one }SiO_2\text{NP}}$ = the ratio of the Au number with a SiO_2NP

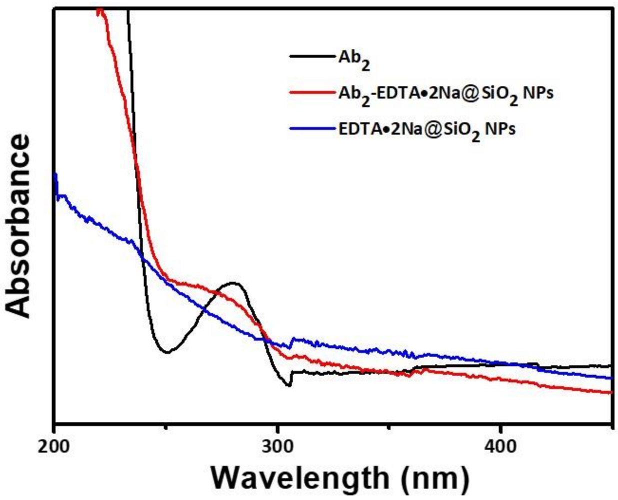

Figure S2. The UV-vis absorption spectra of Ab_2 (black line), EDTA•2Na @SiO_2 NPs (blue line) and the purified Ab_2-EDTA•2Na@SiO_2 NPs bioconjugates (red line).

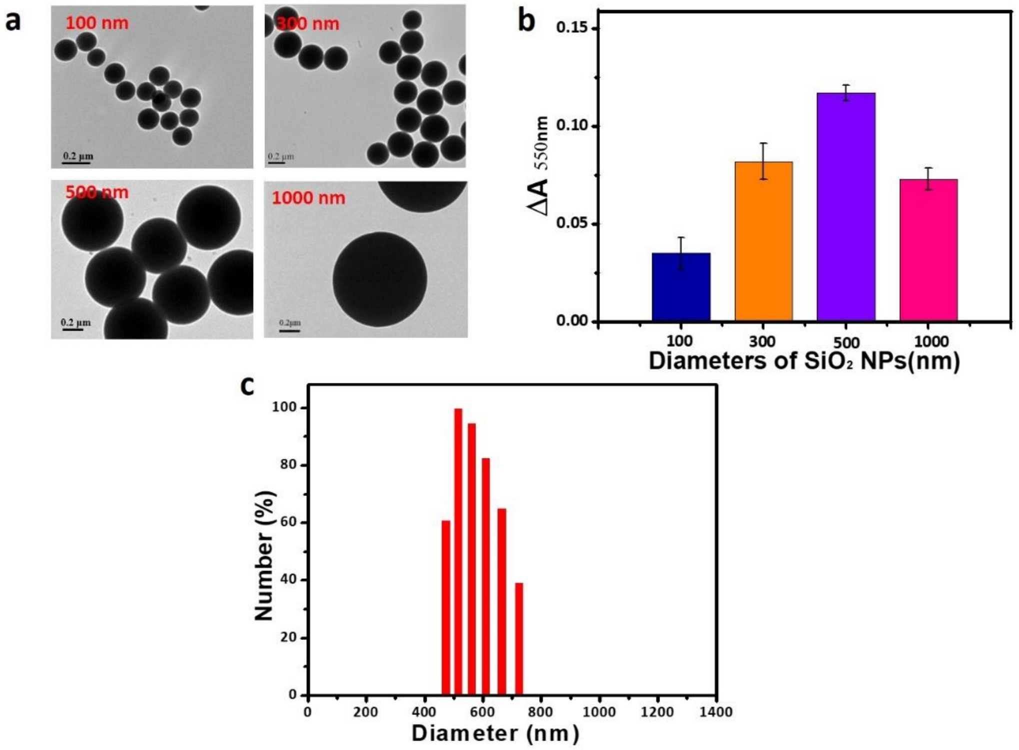

Figure S3. Investigation the effect of diameters of SiO_2 NPs on the detection sensitivity of HBsAg. a) TEM images of SiO_2 NPs with different diameters (100 nm, 300 nm, 500 nm and 1000 nm). b) ΔA value of the generated AuNPs by Scadge-Diag. c) The size distribution of SiO_2 NPs (500 nm) by dynamic light scattering. The results showed that the SiO_2 NPs homogeneously dispersed in the solution and the real size of the SiO_2 NPs was 561.3±48 nm. 500 nm-sized was just used as a code for an easy recognition.

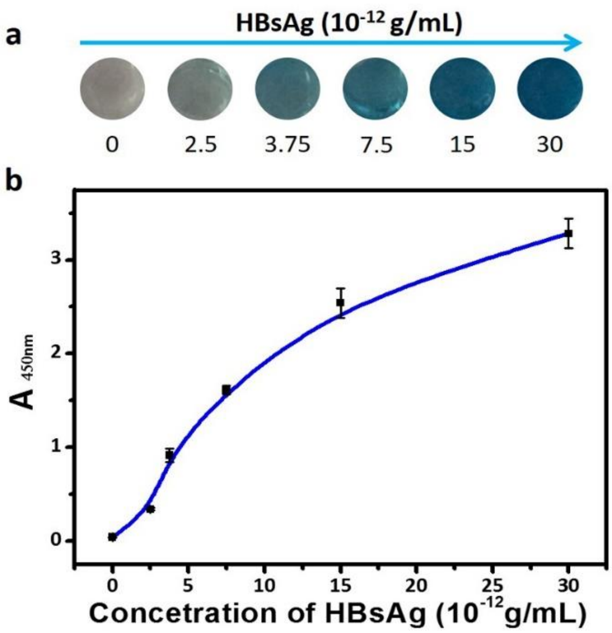

Figure S4. Conventional HRP based a commercial enzyme-linked immunosorbent assay (ELISA) kit for quantitative determination of HBsAg. a) Representative photographs taken from the detection solutions of HBsAg with various concentrations (0, 2.5×10^{-12} g/mL, 3.75×10^{-12} g/mL, 7.5×10^{-12} g/mL, 15×10^{-12} g/mL, 30×10^{-12} g/mL). b) Corresponding plots of A_{450nm} values *versus* different concentrations of HBsAg in ELISA with a limit of detection of 2.5×10^{-12} g/mL.

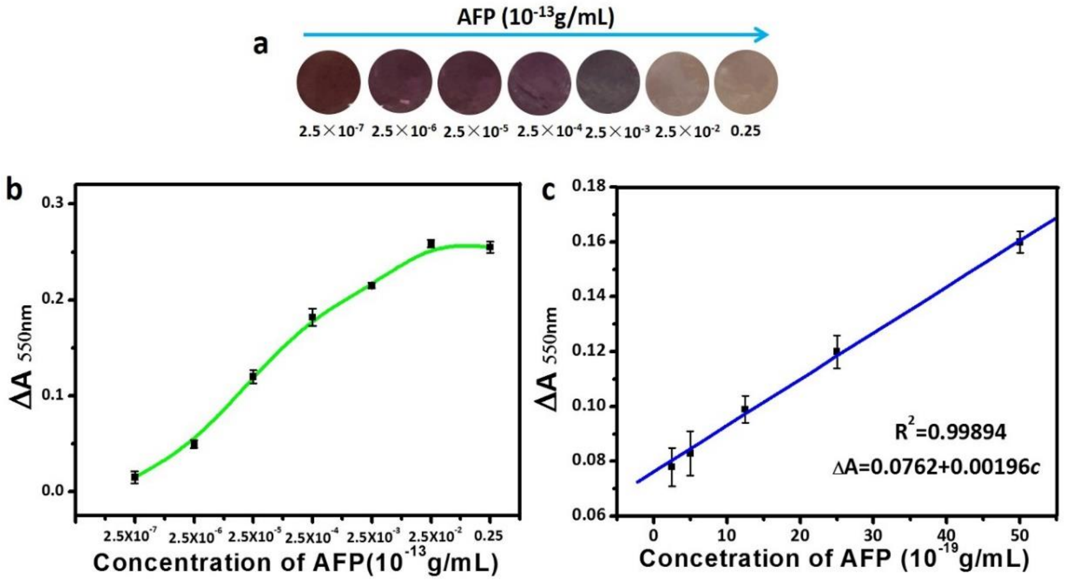

Figure S5. Detection of AFP by Scadge-Diag platform. a) Tonality images of detection solutions with different concentrations of AFP by naked-eye. b) Plotting absorbance values of AuNPs collected at 550 nm *versus* varying concentrations of AFP. c) Calibration curves between the

concentrations of 2.5×10^{-19}~5×10^{-18} g/mL.

Table S1. The cost comparison of HRP and EDTA•2Na obtained from manufacturers abroad and domestic.

Monetary unit: RMB

	SIGMA	Chemart (tianjin) Chemical Technology Co., Ltd.
HRP	1302.21 (100 mg)	190 (10 mg)
EDTA•2Na	315.9 (5 g)	14 (25 g)
